# Postoperative BMI Loss at One Year Correlated with Poor Outcomes in Chinese Gastric Cancer Patients

**DOI:** 10.7150/ijms.46530

**Published:** 2020-08-25

**Authors:** Nan Wang, Jinling Jiang, Wenqi Xi, Junwei Wu, Chenfei Zhou, Min Shi, Chao Wang, Zhenggang Zhu, Jing Liu, Jun Zhang

**Affiliations:** 1 Department of Oncology, Ruijin Hospital, Shanghai Jiao Tong University School of Medicine, No. 197 Ruijin er Road, Shanghai, 200025, China.; 2 Shanghai Institute of Digestive Surgery, Ruijin Hospital, Shanghai Jiao Tong University School of Medicine, No. 197 Ruijin er Road, Shanghai, 200025, China.

**Keywords:** gastric cancer, body mass index, weight loss, prognosis

## Abstract

**Purpose:** The present study focused on the long-term prognostic value of dynamic body mass index (BMI) change in gastric cancer patients who underwent gastrectomy.

**Methods**: Clinical data from a total of 576 gastric cancer patients who underwent radical gastrectomy were collected. Univariate and multivariate analyses were performed to demonstrate the association between dynamic BMI variables (BMI before surgery, 1 month, 6 months or 12 months after surgery) and prognosis (DFS and OS). The correlation between BMI loss after surgery and survival outcomes was also evaluated.

**Results**: Post-operative BMI, especially BMI at one year after surgery (*p*<0.001), was an independent risk factor of recurrence and mortality, wherein patients with high-BMI (≥23) showed significantly better outcomes than patients with normal-BMI (18.5-23) (DFS, HR:0.49; 95% CI:0.31-0.78; OS, HR:0.30; 95% CI: 0.15-0.59). On the contrary, low-BMI (<18.5) patients presented with worse outcomes (DFS, HR: 1.34; 95% CI: 1.00-1.80; OS, HR: 1.68; 95% CI: 1.20-2.34). In addition, compared with moderate BMI loss (≤10%), severe postoperative BMI loss (>10%) at one year was independently associated with substantially worse prognosis for DFS (HR: 1.54; 95% CI: 1.15-2.08) and OS (HR: 1.45; 95% CI: 1.02-2.06). Subgroup analysis indicated that gender (*p*=0.03), extent of resection (*p*<0.001), tumor site (*p*=0.001) and perineural invasion (*p*=0.007) were associated with postoperative BMI loss at one year. The prognostic value of postoperative BMI loss at one year was consistent among most clinicopathological subgroups, except for tumor site (interaction *p*=0.025 for OS).

**Conclusion**: In Chinese gastric cancer patients who underwent gastrectomy, higher postoperative BMI (≥ 23) was significantly associated with longer survival time, whereas severe BMI loss (>10%) at one year after surgery was associated with worse outcomes. Thus, body weight maintenance after treatment is important, and dynamic monitoring of body weight and nutritional status should be emphasized in clinical practice.

## Introduction

Gastric cancer (GC) is a heavy disease burden in China, accounting for about 45% of stomach cancer-related deaths worldwide^1^, and with a 5-year relative survival rate of 35.9% from 2010 to 2014^2^. Since gastric cancer is a highly heterogeneous disease, an improved understanding of risk factors associated with survival outcomes is quite important.

It has been reported that excess body weight accounted for approximately 3.9% of all cancers (544,300 cases)^3^. Obesity is an established risk factor for at least 17 different types of cancers, and portends less favorable outcomes in several cancers 4-6. However, higher body mass index (BMI) is also associated with improved outcomes in some cancers, such as metastatic melanoma, colorectal cancer, and kidney cancer, etc.^7^-^9^, a phenomenon called the “obesity paradox” which is in contrast to the traditional belief that higher BMI is associated with an increased risk of death in the general population.

In GC, it has been reported that increased BMI was positively correlated with the risk of cardia carcinoma^3^, ^10^, however, the relationship between BMI and the risk of GC in other parts of the stomach is still unclear 11, 12. Some studies showed that obesity was associated with diffuse-type adenocarcinoma and presented an increased risk of precancerous conditions in females, potentially due to chronic inflammation in adipose tissue^13^. In addition, the association between BMI and outcomes of GC patients is controversial. Most patients who underwent gastrectomy and chemotherapy suffer body weight loss due to decreased food intake and medication-induced adverse effects^14^. Several retrospective cohort studies showed that the survival outcomes of overweight GC patients were superior to those of underweight GC patients^15^, ^16^, but some studies showed opposite results^17^. Most of the BMI data were measured at diagnosis, few studies have established the relationship between BMI dynamic changes upon therapy and long-term survival in GC patients, particularly in the Chinese population.

In the present study, we aimed to evaluate the relationship between BMI variation at four different time points (before surgery, 1 month, 6 months or 12 months after surgery) and survival outcomes of GC patients.

## Patients and methods

### Study design and cohort populations

This retrospective cohort study was conducted using data from Hospital Information System of Shanghai Ruijin Hospital from February 2010 to October 2017. We collected clinical data from patients with pStage I-III gastric adenocarcinoma who underwent curative gastrectomy (R0 resection) with standard lymphadenectomy (D2 dissection according to Japanese GC Treatment Guideline Version 5) and consecutive adjuvant chemotherapy. Patients with distant metastasis at diagnosis of GC, undergoing neoadjuvant chemotherapy or radiotherapy, not received or not completed adjuvant chemotherapy after operation, undergoing R1 or R2 resection, or patients who died from surgery complications were excluded. Patients with missing preoperative or one-month postoperative BMI data, or missing survival data (n=16) were also excluded. Finally, a total of 576 cases were included in the cohort of Pre BMI (measured 3-5 days before surgery) and Post_1m BMI (measured 4 weeks ±1 week after surgery). A total of 548 cases were included in the cohort of Post_6m BMI (measured 6 months±2 weeks after surgery), 443 cases in the cohort of Post_12m BMI (measured 12 months±2 weeks after surgery). Among them, 19 patients died within one year after surgery, 28 cases of Post_6m BMI data and 86 cases of Post_12m BMI data were missing.

### Definition of variables and Data collection

#### Clinical variables and Outcome assessment

Information was available for all patients on gender, age at diagnosis (≤60; >60), extent of resection (total gastrectomy; subtotal gastrectomy), tumor site (cardia/fundus; body/angulus; antrum/pylorus), pathological tumor type (adenocarcinoma including papillary or tubular adenocarcinoma; mucinous adenocarcinoma or signet-ring cell carcinoma), differentiation degree of tumor (G1/G2; G3), tumor size (maximum diameter of tumor≤2 cm; >2 cm and ≤5 cm; >5 cm), pTNM stage according to the American Joint Committee on Cancer 8th edition, lymphovascular/perineural invasion (yes; no), adjuvant chemotherapy (mono-chemotherapy; combinational chemotherapy). After surgery, follow-up consisted of blood tests, physical examinations and imaging examinations (chest, abdomen and pelvic CT or MRI scan with contrast) every 3 months in the first year, every 6 months in the second year, then annually from 3 to 5 years; Endoscopy was performed annually. Data on overall survival (OS) was obtained from telephone interviews or Shanghai Center for Disease Control and Prevention. OS was calculated from the date of operation until the date of death from any cause. Disease free survival (DFS) was calculated from operation to first observation of disease recurrence through clinical and imaging examinations or death due to any cause. The survival and recurrence status of the patients was last updated in October 2018.

#### Body mass index

Height and weight of patients were measured by trained nurses at each medical visit. BMI was computed in kilograms per height in meter squared. Post_1m/ Post_6m/ Post_12m BMI loss (%) was defined as (Pre-BMI minus Post_1m/ Post_6m/ Post_12m BMI) divided by Pre-BMI.

Due to the large difference in BMI between eastern and western patients, we categorized BMI using Asian-specific criteria[18]as follows: underweight, BMI <18.5; normal weight, BMI 18.5 to <23.0; overweight, BMI 23.0 to <25.0; and obese, BMI ≥25.0. Because there were only 25 (5.6%) cases with BMI > 25.0 in the cohort of Post_12m BMI, we incorporated them into the group of BMI ≥ 23.0. So the patients in our cohorts were assigned into low-BMI group (<18.5), normal-BMI group (18.5 to<23.0) and high-BMI group (≥23.0).

Optimal cutoff values for BMI loss variables were calculated by maximally selected rank statistics[19]using the 'maxstat' package in R Programming Language. For DFS and OS at three time points, they were 11%, 11%, 10%, 7%, 13% and 15%, respectively. BMI loss of 10% is considered clinical meaningful for use, so we chose 10% as the universal cutoff value to classify BMI loss into two categories: ≤10% (moderate loss) and >10% (severe loss).

### Statistical analysis

Socio-demographic and clinicopathologic data were summarized using descriptive statistics. Survival curves for DFS and OS across BMI categories were generated using the Kaplan-Meier method with log-rank tests. To evaluate the effect of BMI and other variables on prognosis, univariate and multivariate analyses were performed using Cox's proportional hazards model. The variables with a *p*-value of 0.05 or less in univariate analysis were pooled in the multivariate analysis. Chi-square test or Fisher's exact test was used to test the association between categorical variables. Subgroup analyses were done using Cox's proportional hazards model, and the interaction test was also conducted. Patients who did not reach a specific endpoint were censored at time of last follow-up. All statistical procedures were performed using SPSS version 24.0 and R language version 3.6.1. A *p*-value of less than 0.05 was considered to be statistically significant.

## Results

### Patients' baseline and BMI characteristics

Of the 576 GC patients, 274 (47.6%) relapsed and 210 (36.5%) died, with a median follow-up of 49.2 months (range: 9.8-109.9). The median age at diagnosis of GC was 59 years old (range: 19~85). The baseline characteristics are depicted in **Table 1.** The distributions of BMI at four time points and three BMI change variables are shown in **Table 2.** Median Pre-BMI was 22.9 kg/m^2^ (range: 14.9~33.7). Median Post_1m BMI was 20.8 kg/m^2^ (range: 13.2~30.1). Median Post_1m BMI loss was 9.1% (range: -32.7%~32.0%), in which 233 (40.5%) patients had severe BMI loss. Median Post_6m BMI was 20.2 kg/m^2^ (range: 13.7~32.1). Median Post_6m BMI loss was 12.0% (range: -20.0%~41.6%), and 325 (59.3%) patients had severe BMI loss. Median Post_12m BMI was 20.2 kg/m^2^ (range: 13.6~29.4). Median Post_12m BMI loss was 11.9% (range: -22.6%~44.6%), and 256 (57.8%) patients had severe BMI loss.

### Correlation between BMI variables and DFS & OS

In univariate analysis, extent of resection, tumor size, pT-stage, pN-stage, pTNM-stage, lymphovascular/perineural invasion and all BMI variables except Pre-BMI were associated with DFS. Age at diagnosis, extent of resection, differentiation degree, tumor size, pT-stage, pN-stage, pTNM-Stage, lymphovascular/perineural invasion and all BMI variables except Post_1m/Post_6m BMI loss were associated with OS. Especially for Post_12m BMI, compared with normal-BMI group (≥18.5 and <23), low-BMI group (BMI <18.5) showed significantly worse outcomes (DFS, HR: 1.75; 95% CI: 1.32-2.32; OS, HR: 2.09; 95% CI: 1.52-2.88), and high-BMI (≥23) showed significantly better outcomes (DFS, HR: 0.57; 95% CI: 0.35-0.88; OS, HR: 0.36; 95% CI: 0.18-0.69). Relative to moderate Post_12m BMI loss (≤10%), severe Post_12m BMI loss (>10%) showed worse survival outcomes (DFS, HR: 1.79; 95% CI: 1.35-2.38; OS, HR: 1.94, 95% CI: 1.39-2.70)** (Table 2).**

**Figure 1:**
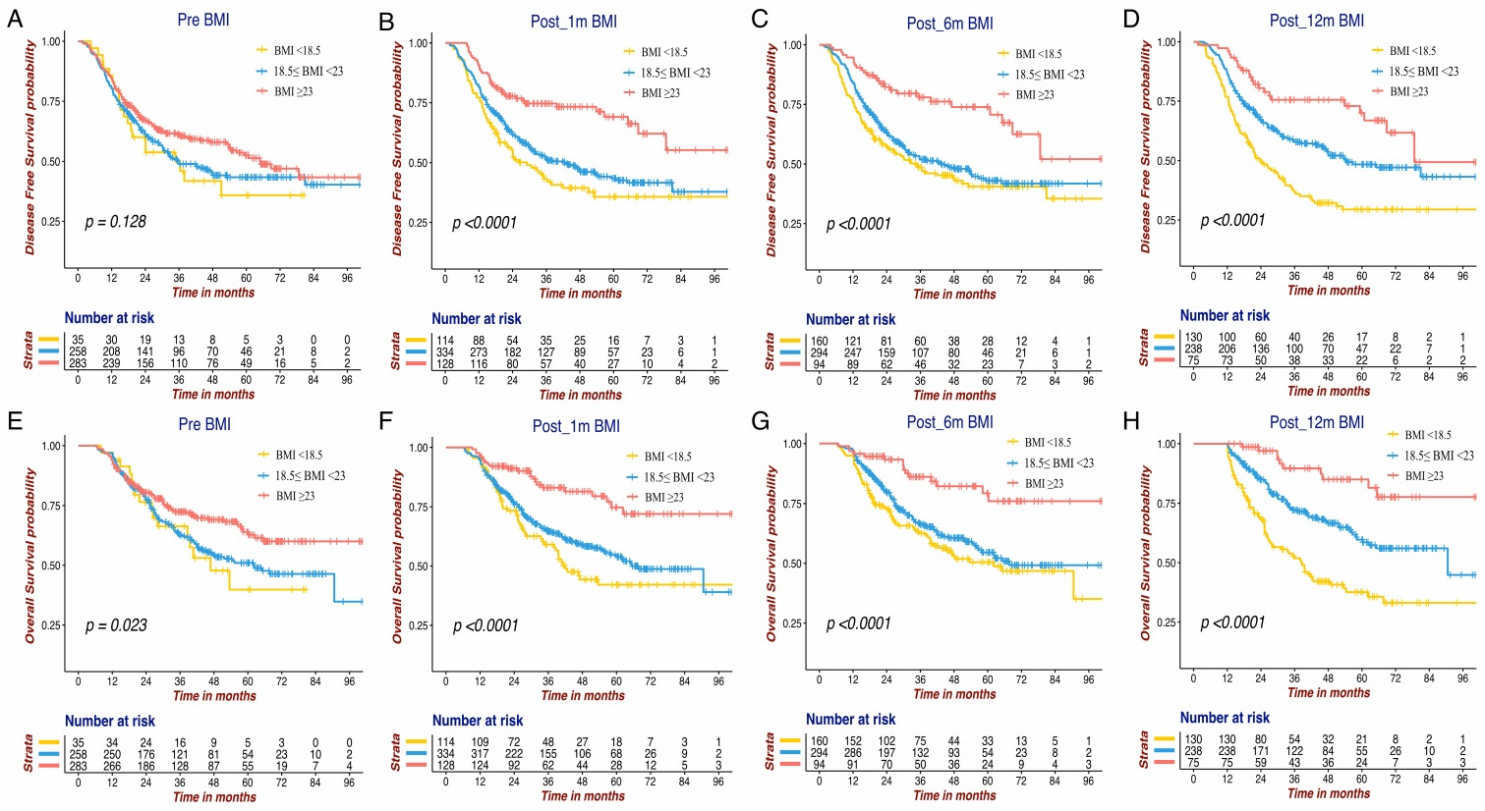
The Kaplan-Meier survival curves of DFS and OS for BMI variables at four different time points in GC patients. DFS of Pre BMI (A), Post_1m BMI (B), Post_6m BMI (C) and Post_12m BMI (D); OS of Pre BMI (E), Post_1m BMI (F), Post_6m BMI (G) and Post_12m BMI (H). DFS: disease free survival; OS: overall survival; BMI: body mass index; GC: gastric cancer.

**Figure 2:**
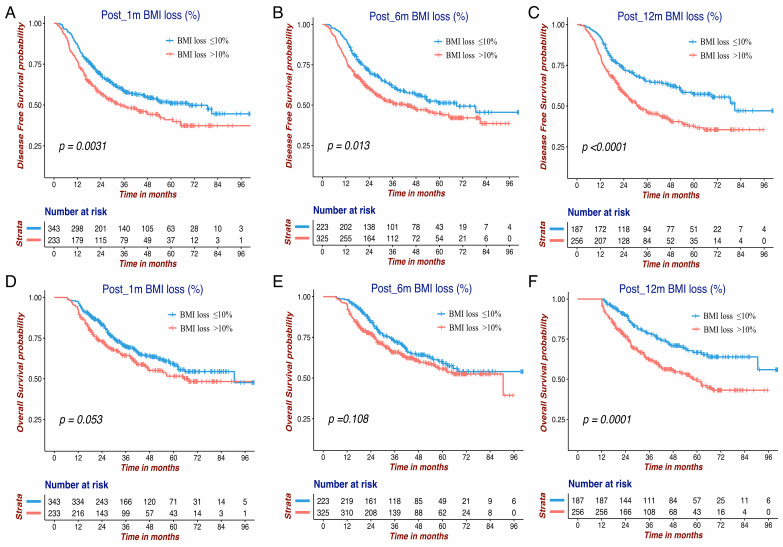
The Kaplan-Meier survival curves of DFS and OS for three BMI change variables in GC patients. DFS of Post_1m BMI loss (A), Post_6m BMI loss (B) and Post_12m BMI loss (C); OS of Post_1m BMI loss (D), Post_6m BMI loss (E) and Post_12m BMI loss (F). DFS: disease free survival; OS: overall survival; BMI: body mass index; GC: gastric cancer.

The Kaplan-Meier survival curves of DFS and OS for the BMI variables are presented in **Figure 1 and Figure 2**. BMI at four time points were all significantly associated with OS. Except for Pre-BMI (*p*=0.128), the other three BMI variables were significantly associated with DFS. As shown in **Figures 1 and 2**, the gap between three BMI group curves became more significant over time. BMI loss variables were all significantly associated with DFS, whereas only Post_12m BMI loss was correlated with OS (*p*=0.0001). Median DFS/OS, 1-year/3-year DFS rate and 3-year/5-year OS rate in different BMI cohorts are listed in **Table S1.** Briefly, the 3-year DFS and 5-year OS in patients with high-BMI were significantly higher than those with low-BMI, particularly for Post_12m BMI (73.9% vs. 35.9%, 81.6% vs. 35.6%). The outcomes of patients with moderate Post_12m BMI loss were much better than those with severe BMI loss (64.6% vs.45.8%, 65.4% vs.48.5%).

**Table 3** shows the multivariate analysis of DFS and OS in the Post_12m cohort, and the analyses of the other three cohorts are shown in **Table S2**. In the Post_12m cohort, low-BMI patients (HR:1.34; 95%CI:1.00-1.80), high-BMI patients (HR:0.49; 95% CI:0.31-0.78), severe Post_12m BMI loss (HR:1.54; 95% CI:1.15-2.08), pT-stage (T4, HR:1.47; 95%CI:1.03-2.08), pN-stage (N3, HR:2.23; 95%CI:1.65-3.01), and perineural invasion (No, HR:0.67; 95%CI, 0.50-0.89) were independent prognostic factors for DFS. Similarly, Post_12m low-BMI (HR:1.68; 95%CI:1.20-2.34), post_12m high-BMI (HR:0.30; 95%CI:0.15-0.59), severe Post_12m BMI loss (HR:1.45; 95%CI:1.02-2.06), pT-stage(T4, HR:1.85; 95%CI: 1.20-2.85), pN-stage (N3, HR:2.52; 95%CI: 1.78-3.56), perineural invasion (No, HR:0.68; 95%CI: 0.48-0.95) and age at diagnosis(>60, HR:1.47; 95%CI: 1.07-2.01) were independent prognostic factors for OS. Post_12m BMI loss was an independent poor risk factor for both DFS and OS, while Post_1m and Post_6m BMI loss showed no significant correlations with outcomes.

### Association between clinical characteristics and BMI variables

As shown in **Table 4**, Pre BMI, Post_12m BMI and Post_12m BMI loss significantly differed between male and female patients. Post_12m BMI was associated with extent of resection (*p*=0.024), tumor site (*p*=0.036) and pN stage (*p*=0.021). Extent of resection (*p*<0.001), tumor site (*p*=0.001) and perineural invasion(*p*=0.007) were also associated with Post_12m BMI loss.

### Prognostic value of Post_12m BMI loss in clinicopathological subgroups

**Figure 3:**
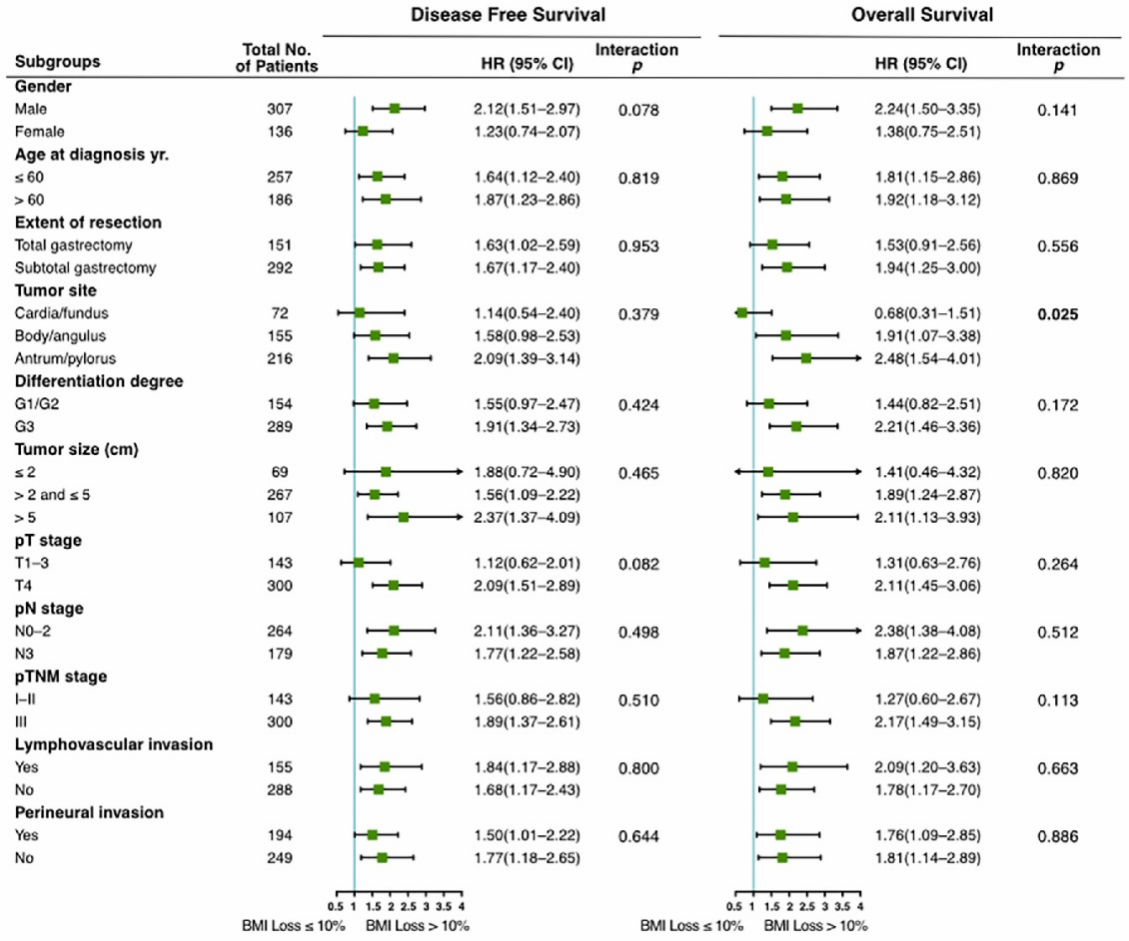
Prognostic value of Post_12m BMI loss in clinicopathological subgroups (severe loss (>10%) vs. moderate loss (≤10%)). BMI: body mass index; HR: hazard ratio; 95% CI: 95% confidence interval.

Subgroup analyses were performed to investigate the consistency of the prognostic value of Post_12m BMI loss in patients with different clinicopathological characteristics. As shown in **Figure 3**, severe BMI loss was associated with worse prognosis than moderate loss in the majority of subgroups. There was no interaction between Post_12m BMI loss and any of clinicopathological factors, except for tumor site (interaction *p*=0.025 for OS). The effect of Post_12m BMI loss on OS in patients with gastric fundus/cardia carcinoma is uncertain and needs to be further explored.

## Discussion

The results of the present study indicated that post-operative BMI variables were independent risks of GC recurrence and mortality. Compared with a relatively stable BMI, severe Post_12m BMI loss (>10%) was independently associated with worse prognosis for DFS and OS, while BMI loss within six months after surgery showed no significant association with survival. The prognostic value of Post_12m BMI loss was consistent among the majority of clinicopathological subgroups. These data indicate that short-term weight loss after surgery may not affect survival, but long-term maintenance of body weight is more significant. We also found that the time point of 12 months after surgery is more valuable for body weight evaluation, which avoids the influence of surgery or adjuvant chemotherapy on BMI and represents patients' stable long-term nutritional status.

Studies on BMI and GC survival differ in terms of BMI assessment, adjustment, inclusion and exclusion criteria. Some studies indicated that overweight and obesity at diagnosis were adverse prognostic characteristics as too much abdominal fat led to unsuccessful and incomplete lymph node dissection 20-22. In addition, a higher BMI was associated with longer duration of operation and increasing risk of postoperative complications 23. The presence of comorbid diseases associated with being overweight may also predict poor survival. Chen et al. 15 reported that GC patients with high-BMI exhibited a significantly prolonged OS compared with underweight patients, which was consistent with our research. The author explained that tumor stage was an important factor, since the percentage of advanced cancers was doubled in low-BMI patients compared to high-BMI patients 15. In our study, all enrolled patients underwent gastrectomy (R0 resection) with standard lymphadenectomy (D2), and patients who died from surgical complications were excluded. BMI distribution between stages was well balanced. Furthermore, we found that severe BMI loss (>10%) at one year after surgery was an independent risk factor for both OS and DFS, which was similar to previous reports that postoperative severe BMI loss (>4.5) (HR, 1.79; 95% CI, 1.29-2.50) was associated with higher mortality^16^, and body weight loss ≥15% at 1 month after gastrectomy might lead to poor survival^24^**.** Compared to previous research that was limited to either one certain time point after gastrectomy or an uncertain wide time range, our study focused on 4 precise time points (pre, post_1m, post_6m, post_12m) and emphasized the importance of BMI dynamic monitoring. We also pointed out the most important time point was 12 months after surgery. These results were rarely mentioned in previous studies.

There are several potential interpretations for our results. First, due to characteristics of GC and Chinese physique, few patients were excessively obese, which could reduce some confounding factors like operation difficulty. Second, patients with higher BMI had decreased treatment-related toxicity and increased response to anti-cancer therapy 25, 26. Meyerhardt's study showed that overweight patients had a lower rate of Grade 3-4 leukopenia and any toxicity ≥Grade 3, and could tolerate actual weight-based doses of chemotherapy 26, while underweight patients often displayed higher rate of withdrawal or reduction of chemotherapy 27. Furthermore, overweight or obesity and a stable BMI status might function as protective factors from malnutrition, cancer cachexia, or altered immune functions, which greatly influence the prognosis of cancer patients 28, 29. Wang et al. demonstrated that obesity increased T cell aging which resulted in higher PD-1 expression and dysfunction driven by leptin signaling 30, which provided an explanation for a recent report that obese patients with melanoma showed improved outcomes with immunotherapy 7.

A novel aspect of our study included the assessment of longitudinal BMI and dynamic BMI changes at four different time points, which has rarely been investigated before, particularly in the Chinese population. We demonstrated that one year after surgery is the most valuable time point for BMI evaluation. As an easily available clinical parameter, BMI before and after surgery could add additional prognostic information for GC patients.

Our analysis still has some limitations. As a single-center retrospective study, it is difficult to avoid confounding factors and bias. Besides, though BMI is a widely used parameter for obesity, it is an imperfect surrogate of adiposity and may misclassify body composition. Li et al reported that visceral and subcutaneous fat might be new independent predictive factors of survival in locally advanced gastric carcinoma patients 31. Finally, our research only indicates a correlation between postoperative BMI and prognosis, but cannot prove there is a causal relationship between them.

In conclusion, our data suggest that in GC patients, higher BMI (>23) is associated with improved survival, whereas remarkable BMI loss (>10%) is significantly correlated with worse prognosis, particularly at one year after surgery. Monitoring body weight loss as a prognostic factor after treatment should be emphasized during follow up. Since our study is retrospective and exploratory, the results need to be further validated in a multi-center prospective study.

## Supplementary Material

Supplementary tables.

## Figures and Tables

**Table 1:** Baseline characteristics and univariate analysis of DFS and OS (*N=576*)

Patients characteristics	N (%)	DFS		OS	
		HR (95%CI)	*p* value	HR (95%CI)	*p* value
**Gender**			0.319		0.198
Male	400(69.4)	1		1	
female	176(30.6)	0.88(0.68-1.14)		0.82(0.61-1.11)	
**Age at diagnosis yr.**			0.063		**0.023**
≤ 60	321(55.7)	1		1	
> 60	255(44.3)	1.25(0.99-1.59)		1.37(1.04-1.80)	
**Extent of resection**			**<0.001**		**<0.001**
Total gastrectomy	204(35.4)	1		1	
Subtotal gastrectomy	372(64.6)	0.60(0.48-0.77)		0.52(0.40-0.69)	
**Tumor site**			0.538		0.762
Cardia/fundus	99(17.2)	1		1	
Body/angulus	203(35.2)	1.05(0.74-1.47)		0.99(0.66-1.48)	
Antrum/pylorus	274(47.6)	0.90(0.65-1.26)		0.89(0.61-1.32)	
**Pathological tumor type**			0.811		0.408
Adenocarcinoma^ a^	502(87.2)	1		1	
Mucinous adenocarcinoma or signet-ring cell carcinoma	74(12.8)	0.96(0.67-1.37)		1.18(0.80-1.73)	
**Differentiation degree**			0.115		**0.033**
G1/G2	207(35.9)	1		1	
G3	369(64.1)	1.22(0.95-1.57)		1.37(1.02-1.84)	
**Tumor size(cm)^b^**			**<0.001**		**<0.001**
≤2	89(15.5)	1		1	
>2 and ≤5	342(59.4)	2.09(1.36-3.20)		2.37(1.41-3.99)	
>5	145(25.2)	2.74(1.74-4.31)		3.36(1.95-5.81)	
**pT stage**			**<0.001**		**<0.001**
T1-3	187(32.5)	1		1	
T4	389(67.5)	1.86(1.40-2.47)		2.40(1.70-3.39)	
**pN stage**			**<0.001**		**<0.001**
N0-2	335(58.2)	1		1	
N3	241(41.8)	2.88(2.26-3.67)		3.13(2.36-4.13)	
**pTNM stage**			**<0.001**		**<0.001**
I-II	177(30.7)	1		1	
III	399(69.3)	2.25(1.67-3.02)		3.12(2.14-4.54)	
**Lymphovascular invasion**			**<0.001**		**<0.001**
Yes	213(37)	1		1	
No	363(63)	0.53(0.42-0.68)		0.54(0.41-0.71)	
**Perineural invasion**			**<0.001**		**<0.001**
Yes	255(44.3)	1		1	
No	321(55.7)	0.55(0.43-0.69)		0.52(0.40-0.69)	
**Adjuvant chemotherapy**			0.520		0.477
Mono-chemotherapy	67(11.6)	1		1	
Combinational chemotherapy	509(88.4)	0.89(0.62-1.28)		0.86(0.57-1.30)	

^a^ including papillary or tubular adenocarcinoma; ^b^ maximum diameter of tumor DFS: disease free survival; OS: overall survival; HR: hazard ratio; 95% CI: 95% confidence interval; BMI: body mass index

**Table 2:** Univariate analysis of BMI variables for DFS and OS

BMI variables	N (%)	DFS		OS	
		HR (95%CI)	*p* value	HR (95%CI)	*p* value
**Pre BMI (kg/m^2^)**	**N=576**		0.128		**0.023**
<18.5	35(6.1)	1.13(0.71-1.80)		1.12(0.66-1.90)	
18.5 to <23.0	258(44.8)	1		1	
≥23	283(49.1)	0.78(0.63 -1.02)		0.70(0.52-0.92)	
**Post_1m BMI (kg/m^2^)**	**N=576**		**<0.001**		**<0.001**
<18.5	114(19.8)	1.25(0.94-1.66)		1.29(0.94-1.78)	
18.5 to <23.0	334(58)	1		1	
≥23	128(22.2)	0.49(0.35-0.71)		0.42(0.27-0.66)	
**Post_1m BMI loss (%)**	**N=576**		**0.003**		0.053
≤10%	343(59.5)	1		1	
>10%	233(40.5)	1.43(1.13-1.81)		1.33(1.00-1.74)	
**Post_6m BMI (kg/m2)**	**N=548**		**<0.001**		**<0.001**
<18.5	160(29.2)	1.18(0.91-1.54)		1.24(0.92-1.67)	
18.5 to <23.0	294(53.6)	1		1	
≥23	94(17.2)	0.46(0.29-0.70)		0.38(0.22-0.65)	
**Post_6m BMI loss (%)**	**N=548**		**0.013**		0.108
≤10%	223(40.7)	1		1	
>10%	325(59.3)	1.38(1.07-1.77)		1.27(0.95-1.69)	
**Post_12m BMI (kg/m2)**	**N=443**		**<0.001**		**<0.001**
<18.5	130(29.3)	1.75(1.32-2.32)		2.09(1.52-2.88)	
18.5 to <23.0	238(53.7)	1		1	
≥23	75(16.9)	0.57(0.35-0.88)		0.36 (0.18-0.69)	
**Post_12m BMI loss (%)**	**N=443**		**<0.001**		**<0.001**
≤10%	187(42.2)	1		1	
>10%	256(57.8)	1.79(1.35-2.38)		1.94(1.39-2.70)	

Pre BMI: measured before surgery; Post_1m BMI: measured 4 weeks ±1 week after surgery; Post_6m BMI: measured 6 months±2 weeks after surgery; Post_12m BMI: measured 12 months±2 weeks after surgery; DFS: disease free survival; OS: overall survival; HR: hazard ratio; 95% CI: 95% confidence interval; BMI: body mass index

**Table 3:** Multivariate analysis of DFS and OS in the cohort of Post_12m BMI

Variables	DFS		OS	
	HR (95%CI)	*p* value	HR (95%CI)	*p* value
**Post_12m BMI (kg/m2)**		**<0.001**		**<0.001**
< 18.5	1.34(1.00-1.80)		1.68(1.20-2.34)	
18.5 to <23.0	1		1	
≥ 23	0.49(0.31-0.78)		0.30(0.15-0.59)	
**Post_12m BMI loss (%)**		**0.004**		**0.039**
≤ 10%	1		1	
> 10%	1.54(1.15-2.08)		1.45(1.02-2.06)	
**pT stage**		**0.032**		**0.005**
T1-3	1		1	
T4	1.47(1.03-2.08)		1.85(1.20-2.85)	
**pN stage**		**<0.001**		**<0.001**
N0-2	1		1	
N3	2.23(1.65-3.01)		2.52(1.78-3.56)	
**Differentiation degree**	NI			0.644
G1/G2			1	
G3			0.92(0.66-1.30)	
**Lymphovascular invasion**		0.201		0.625
Yes	1		1	
No	0.83(0.62-1.11)		0.92(0.65-1.30)	
**Perineural invasion**		**0.006**		**0.024**
Yes	1		1	
No	0.67(0.50-0.89)		0.68(0.48-0.95)	
**Age at diagnosis yr.**	NI			**0.016**
≤ 60			1	
> 60			1.47(1.07-2.01)	

Post_12m BMI: measured 12 months±2 weeks after surgery; Pre BMI: measured before surgery; DFS: disease free survival; OS: overall survival; HR: hazard ratio; 95% CI: 95% confidence interval; BMI: body mass index; NI: not include

**Table 4:** Association between clinical characteristics and Pre BMI, Post_12m BMI, Post_12m BMI loss

Clinical characteristics	Pre BMI(n=576)			*p* value	Post_12m BMI(n=443)			*p* value	Post_12m BMI loss (n=443)		*p* value
	<18.5	18.5 to <23.0	≥23		<18.5	18.5 to <23.0	≥23		≤10%	>10%	
**Gender**				**<0.001**				**<0.001**			**0.030**
Male	17(4.3%)	163(40.8%)	220(55%)		69(22.5%)	181(59%)	57(18.6%)		140(45.6%)	167(54.4%)	
female	18(10.2%)	95(54%)	63(35.8%)		61(44.9%)	57(41.9%)	18(13.2%)		47(34.6%)	89(65.4%)	
**Age at diagnosis yr.**				0.280				0.811			0.768
≤ 60	23(7.2%)	148(46.1%)	150(46.7%)		75(29.2%)	136(52.9%)	46(17.9%)		110(42.8%)	147(57.2%)	
> 60	12(4.7%)	110(43.1%)	133(52.2%)		55(29.6%)	102(54.8%)	29(15.6%)		77(41.4%)	109(58.6%)	
**Extent of resection**				0.067				**0.024**			**<0.001**
Total gastrectomy	16(7.8%)	79(38.7%)	109(53.4%)		55(36.4%)	78(51.7%)	18(11.9%)		46(30.5%)	105(69.5%)	
Subtotal gastrectomy	19(5.1%)	179(48.1%)	174(46.8%)		75(25.7%)	160(54.8%)	57(19.5%)		141(48.3%)	151(51.7%)	
**Tumor site**				0.412				**0.036**			**0.001**
Cardia/fundus	7(7.1%)	39(39.4%)	53(53.5%)		25(34.7%)	41(56.9%)	6(8.3%)		18(25%)	54(75%)	
Body/angulus	16(7.9%)	91(44.8%)	96(47.3%)		50(32.3%)	84(54.2%)	21(13.5%)		63(40.6%)	92(59.4%)	
Antrum/pylorus	12(4.4%)	128(46.7%)	134(48.9%)		55(25.5%)	113(52.3%)	48(22.2%)		106(49.1%)	110(50.9%)	
**Pathological tumor type**				0.727				0.882			0.185
Adenocarcinoma ^a^	32(6.4%)	222(44.2%)	248(49.4%)		112(29.3%)	204(53.4%)	66(17.3%)		166(43.5%)	216(56.5%)	
Mucinous adenocarcinoma or signet-ring cell carcinoma	3(4.1%)	36(48.6%)	35(47.3%)		18(29.5%)	34(55.7%)	9(14.8%)		21(34.4%)	40(65.6%)	
**Differentiation degree**				0.818				0.065			0.313
G1/G2	14(6.8%)	90(43.5%)	103(49.8%)		38(24.7%)	82(53.2%)	34(22.1%)		70(45.5%)	84(54.5%)	
G3	21(5.7%)	168(45.5%)	180(48.8%)		92(31.8%)	156(54%)	41(14.2%)		117(40.5%)	172(59.5%)	
**Tumor size(cm)^ b^**				0.444				0.986			0.877
≤2	6(6.7%)	42(47.2%)	41(46.1%)		21(30.4%)	35(50.7%)	13(18.8%)		29(42%)	40(58%)	
>2 and ≤5	17(5%)	159(46.5%)	166(48.5%)		78(29.2%)	145(54.3%)	44(16.5%)		115(43.1%)	152(56.9%)	
>5	12(8.3%)	57(39.3%)	76(52.4%)		31(29%)	58(54.2%)	18(16.8%)		43(40.2%)	64(59.8%)	
**pT stage**				0.542				0.168			0.454
T1-3	13(7%)	88(47.1%)	86(46%)		37(25.9%)	86(60.1%)	20(14%)		64(44.8%)	79(55.2%)	
T4	22(5.7%)	170(43.7%)	197(50.6%)		93(31%)	152(50.7%)	55(18.3%)		123(41.3%)	177(59%)	
**pN stage**				0.550				**0.021**			0.778
N0-2	20(6%)	144(43%)	171(51%)		67(25.4%)	156(59.1%)	41(15.5%)		110(41.7%)	154(58.3%)	
N3	15(6.2%)	114(47.3%)	112(46.5%)		63(35.2%)	82(45.8%)	34(19%)		77(43.0%)	102(57.0%)	
**pTNM stage**				0.660				0.163			0.587
I-II	10(5.6%)	75(42.4%)	92(52%)		35(24.5%)	86(60.1%)	22(15.4%)		63(44.1%)	80(55.9%)	
III	25(6.3%)	183(45.9%)	191(47.9%)		95(31.7%)	152(50.7%)	53(17.7%)		124(41.3%)	176(58.7%)	
**Lymphovascular invasion**				0.878				0.846			0.134
Yes	14(6.6%)	93(43.7%)	106(49.8%)		48(31%)	82(52.9%)	25(16.1%)		58(37.4%)	97(62.6%)	
No	21(5.8%)	165(45.5%)	177(48.8%)		82(28.5%)	156(54.2%)	50(17.4%)		129(44.8%)	159(55.2%)	
**Perineural invasion**				0.900				0.440			**0.007**
Yes	15(5.9%)	112(43.9%)	128(50.2%)		63(32.5%)	100(51.5%)	31(16%)		68(35.1%)	126(64.9%)	
No	20(6.2%)	146(45.5%)	155(48.3%)		67(26.9%)	138(55.4%)	44(17.7%)		119(47.8%)	130(52.2%)	
**Adjuvant Chemotherapy**				0.659				0.767			0.737
Mono-chemotherapy	5(7.5%)	27(40.3%)	35(52.2%)		13(26%)	27(54%)	10(20%)		20(40%)	30(60%)	
Combinational chemotherapy	30(5.9%)	231(45.4%)	248(48.7%)		117(29.8%)	211(53.7%)	65(16.5%)		167(42.5%)	226(57.5%)	

^a^ including papillary or tubular adenocarcinoma; ^b^ maximum diameter of tumor Pre BMI: measured before surgery; Post_12m BMI: measured 12 months±2 weeks after surgery; BMI: body mass index
