# Supplementary material for: Postoperative BMI Loss at One Year Correlated with Poor Outcomes in Chinese Gastric Cancer Patients
Source: Int J Med Sci. 2020 Aug 25;17(15):2276–84. doi: 10.7150/ijms.46530 (PMC7484638; doi:10.7150/ijms.46530)
Supplement: Supplementary file 1 — Supplementary tables. [file ijmsv17p2276s1.pdf]

Table S1. Median and proportion of DFS and OS of BMI characteristics

Variables	N(%)	DFS			OS		
		mDFS (mo.)	1-year DFS (%)	3-year DFS (%)	mOS (mo.)	3-year OS (%)	5-year OS (%)
**Pre BMI (kg/m^2)**	**N=576**						
<18.5	35(6.1)	34.7	80.0	46.0	46.8	61.9	39.8
18.5 to <23.0	258(44.8)	35.6	80.2	48.9	62.1	62.7	49.9
≥23	283(49.1)	65.1	84.5	60.5	NR	71.8	62.7
**Post_1m BMI (kg/m^2)**	**N=576**						
<18.5	114(19.8)	29.1	76.3	42.0	41.9	57.9	NA
18.5 to <23.0	334(58)	43.1	81.1	51.0	65.4	64.6	53.9
≥23	128(22.2)	NR	89.8	73.3	NR	83.1	72.0
**Post_1m BMI loss (%)**	**N=576**						
≤10%	343(59.5)	68.8	86.3	57.8	90.6	69.7	58.4
>10%	233(40.5)	35.3	76.4	48.8	67.7	63.7	50.1
**Post_6m BMI (kg/m^2)**	**N=548**						
<18.5	160(29.2)	34.7	75.0	47.8	62.1	62.5	48.7
18.5 to <23.0	294(53.6)	43.6	83.7	51.9	67.7	66.3	54.5
≥23	94(17.2)	NR	93.6	77.9	NR	84.3	76.0
**Post_6m BMI loss (%)**	**N=548**						
≤10%	223(40.7)	68.8	90.1	60.8	NR	72.4	58.9
>10%	325(59.3)	43.6	78.5	51.1	90.6	65.4	54.6
**Post_12m BMI (kg/m^2)**	**N=443**						
<18.5	130(29.3)	24.1	76.2	35.9	38.4	51.9	35.6
18.5 to <23.0	238((53.7)	54.4	86.6	57.8	90.6	71.6	58.6
≥23	75(16.9)	78.7	96.0	73.9	NR	88.7	81.6
**Post_12m BMI loss (%)**	**N=443**						
≤ 10%	187(42.2)	80.9	92.0	64.6	NR	77.9	65.4
>10%	256(57.8)	31.2	80.5	45.8	58.2	61.8	48.5

Pre BMI: measured before surgery; Post_1m BMI: measured 4 weeks ±1 week after surgery; Post_6m BMI: measured 6 months±2 weeks after surgery; Post_12m BMI: measured 12 months±2 weeks after surgery; Post_1m/ Post_6m/ Post_12m BMI loss (%): (Pre-BMI minus Post_1m/ Post_6m/ Post_12m BMI) divided by Pre-BMI; mDFS: median disease-free survival (month); mOS: median overall survival (month); BMI: body mass index; NR: not reached; NA: not available

Table S2. Multivariate analysis of DFS and OS in Pre / Post_1m / 6m cohorts.

Variables	DFS		OS	
	HR (95%CI)	*p* value	HR (95%CI)	*p* value
**Pre BMI**				
**Pre BMI (kg/m^2)** VS 18.5 to <23.0		0.108		**0.011**
< 18.5	1.14(0.71-1.82)		1.13(0.66-1.91)	
≥ 23	0.79(0.62-1.02)		0.66(0.50-0.88)	
**pT stage V**S T1-3		0.141		**0.013**
T4	1.26(0.93-1.71)		1.60(1.10-2.32)	
**pN stage** VS N0-2		**<0.001**		**<0.001**
N3	2.41(1.86-3.12)		2.54(1.89-3.42)	
**Differentiation degree** VS G1/G2	NI			0.321
G3			1.17(0.86-1.58)	
**Lymphovascular invasion** VS Yes		**0.028**		0.127
No	0.75(0.58-0.97)		0.79(0.59-1.07)	
**Perineural invasion** VS Yes		**0.004**		**0.007**
No	0.68(0.53-0.89)		0.66(0.50-0.88)	
**Age at diagnosis yr.** VS ≤ 60	NI			**0.004**
> 60			1.50(1.14-1.97)	
**Post_1m BMI**				
**Post_1m BMI (kg/m^2)** VS 18.5 to <23.0		**<0.001**		**<0.001**
< 18.5	1.20(0.89-1.59)		1.36(0.98-1.88)	
≥ 23	0.48(0.34-0.69)		0.40(0.26-0.63)	
**Post_1m BMI loss (%)** VS ≤10%		0.054		0.295
> 10%	1.27(1.0-1.62)		1.16(0.88-1.53)	
**pT stage V**S T1-3		0.106		**0.007**
T4	1.29(0.95-1.74)		1.66(1.15-2.41)	
**pN stage** VS N0-2		**<0.001**		**<0.001**
N3	2.38(1.84-3.10)		2.56(1.89-3.46)	
**Differentiation degree** VS G1/G2	NI			0.678
G3			1.07(0.79-1.45)	
**Lymphovascular invasion** VS Yes		**0.035**		0.133
No	0.76(0.59-0.98)		0.80(0.59-1.07)	
**Perineural invasion** VS Yes		**0.004**		**0.010**
No	0.69(0.53-0.89)		0.68(0.51-0.91)	
**Age at diagnosis yr.** VS ≤ 60	NI			**0.002**
> 60			1.54(1.17-2.03)	

Continued

**Post_6m BMI**				
**Post_6m BMI (kg/m^2)** VS 18.5 to <23.0		**<0.001**		**<0.001**
< 18.5	1.07(0.81-1.40)		1.23(0.90-1.69)	
≥ 23	0.43(0.28-0.66)		0.35(0.20-0.60)	
**Post_6m BMI loss (%)** VS ≤10%		0.080		0.222
> 10%	1.26(0.97-1.64)		1.21(0.89-1.64)	
**pT stage V**S T1-3		0.223		**0.017**
T4	1.22(0.89-1.68)		1.62(1.09-2.40)	
**pN stage** VS N0-2		**<0.001**		**<0.001**
N3	2.49(1.89-3.27)		2.74(2.00-3.76)	
**Differentiation degree** VS G1/G2	NI			0.587
G3			1.09(0.80-1.49)	
**Lymphovascular invasion** VS Yes		0.091		0.317
No	0.79(0.61-1.04)		0.85(0.63-1.16)	
**Perineural invasion** VS Yes		**0.001**		**0.002**
No	0.64(0.49-0.83)		0.61(0.45-0.83)	
**Age at diagnosis yr.** VS ≤ 60	NI			**0.001**
> 60			1.63(1.22-2.18)	

Pre BMI: measured before surgery; Post_1m BMI: measured 4 weeks ±1 week after surgery; Post_6m BMI: measured 6 months±2 weeks after surgery; Post_1m/ Post_6m BMI loss (%): (Pre-BMI minus Post_1m/ Post_6m BMI) divided by Pre-BMI; DFS: disease free survival; OS: overall survival; HR: hazard ratio; BMI: body mass index; NI: not include
